# Diosgenin ameliorates silica‐induced tuberculosis in rats

**DOI:** 10.1002/ame2.70055

**Published:** 2025-06-30

**Authors:** Williams Asamoah Adu, Selase Ativui, Michael Ofori, George Owusu, Cynthia Amaning Danquah, Paul Poku Sampene Ossei

**Affiliations:** ^1^ Department of Pharmaceutical Sciences, Faculty of Applied Science and Technology Sunyani Technical University Sunyani Ghana; ^2^ Department of Pharmacology, Faculty of Pharmacy and Pharmaceutical Sciences College of Health Sciences, Kwame Nkrumah University of Science and Technology, PMB Kumasi Ghana; ^3^ Department of Medical Laboratory Science University of Energy and Natural Resources Sunyani Ghana; ^4^ Department of Pathology, School of Medicine and Dentistry Kwame Nkrumah University of Science and Technology Kumasi Ghana

**Keywords:** antioxidant, diosgenin, mycobacterium, pulmonary fibrosis, silicosis, silicotuberculosis

## Abstract

**Background:**

Silicosis is an occupational lung disease that is caused by chronic exposure to silica dust. Silica‐exposed workers are at higher risk of developing TB, resulting in lung fibrosis and significant respiratory dysfunction. Diosgenin is a steroidal saponin that has been shown to exert a therapeutic effect on lung injury. Therefore, we investigated the potential efficacy of diosgenin in treating silicotuberculosis by evaluating its effectiveness against *Mycobacterium smegmatis*, as well as its antifibrotic and antioxidant effects in silica‐induced TB in rats.

**Methods:**

Silicosis was induced by intratracheal instillation of 50 mg/kg crystalline silica in Sprague–Dawley rats. Rats were grouped into 7 (10 per group). Different doses of diosgenin (1, 10, and 20 mg/kg) and saline were administered for 30 days. Afterwards, five rats from each group were sacrificed, and the five remaining rats in each group, except the control, received *Mycobacterium smegmatis*. Treatment continued until the 50th day, and the animals were sacrificed at the end of the experiment. The result was analyzed using a one‐way analysis of variance (ANOVA) with GraphPad Prism.

**Results:**

At a half‐maximal inhibition concentration of 0.006043 μg/mL, diosgenin inhibited the growth of *Mycobacterium smegmatis*. Oxidative stress markers such as malondialdehyde were significantly reduced. The health‐enhancing effects of catalase and superoxide dismutase were elevated. Additionally, histological findings demonstrated a significant improvement in respiratory function following diosgenin treatment.

**Conclusion:**

Diosgenin treatment inhibited the growth of *Mycobacterium smegmatis,* leading to a reduction in the susceptibility of rats to infection and improved pulmonary function through its antioxidant effect.

## Graphical abstract

Silicosis increases susceptibility to tuberculosis due to impaired macrophage function and oxidative stress. This study demonstrates that diosgenin, a steroidal saponin, significantly inhibits *Mycobacterium smegmatis* (IC₅₀ = 0.006 μg/mL), reduces lung fibrosis, and restores antioxidant enzyme levels (↑SOD, ↑CAT, ↓MDA) in silica‐exposed rats. Histological analysis revealed improved alveolar architecture and reduced inflammation following 30–50 days of treatment. These findings suggest that diosgenin is a promising dual‐action compound for managing silicotuberculosis through antimicrobial, antioxidant, and antifibrotic mechanisms.

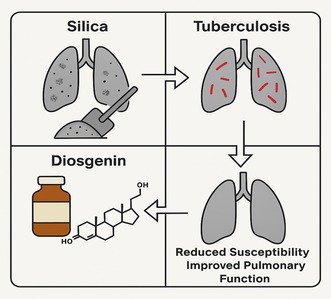

## 1 INTRODUCTION

Silicosis is a work‐related interstitial lung disease caused by the long‐term inhalation of crystalline silica dust.^1^ The prevalence and incidence of this disease have been rising in developing countries such as China, South Africa, Brazil and among underground miners in Ghana.^2^ It is estimated that 10 million workers are exposed to crystalline silica worldwide.^3^ Workers exposed to silica face a threefold or greater risk of developing mycobacterium infection, resulting in a complication known as silicotuberculosis.^4^ Thus, there is a close association between silicosis and tuberculosis.^5^

The anatomy of the respiratory tract exposes the lungs to the toxic effects of dust particles.^6^ Silica can withstand enzymatic breakdown. The inhaled particle is engulfed by an alveolar macrophage, which serves as the first line of defense. Moreover, silica dust resists digestion by the lysosome. Also, the particle size is too small to evade mucociliary clearance in the lungs. Silica irritates the mitochondrial membrane, resulting in increased levels of reactive oxygen species. The mitochondrial membrane potential is eventually impaired, reducing ATP production.^7^ Proinflammatory cytokine levels are elevated to stimulate a profibrotic response, leading to collagen deposition.^8^ The lung is characterized by nodular lesion formation, epithelial‐mesenchymal transition, fibroblast activation to myofibroblast, collagen hyperplasia, and fibrosis formation.^9^ The imbalance between enzymes and levels of free radicals determines the degree of damage. The situation is further worsened by mycobacterium infection.^10^ Once the alveolar macrophage defense is compromised, latent tubercle bacilli are activated, leading to the initiation and progression of TB.

*Mycobacterium tuberculosi*s can thrive within the host's primary immune cells for an extended period under various stress conditions. In addition to its complex structure, *Mycobacterium tuberculosis* is a slow‐growing and highly infectious bacterium. This poses a significant setback in the drug screening procedures for inhibiting mycobacterial growth. It has been reported that *M. smegmatis* is over 90 per cent identical to *M. tuberculosis* and shares a common gene involved in stress adaptation.^11^ A *M. smegmatis* strain (ATCC 607) has been reported to show drug susceptibility similar to that of the *Mycobacterium tuberculosis* H37Rv strain. To avoid the use of highly infectious *M. tuberculosis*, which requires a higher level of safety, *Mycobacterium smegmatis* was used as a surrogate model to study the pattern of the disease.^12^, ^13^

Silicosis is a potentially fatal disease that severely impacts lung function and survival.^14^ Tuberculosis significantly contributes to morbidity and mortality in patients. Currently, there is no effective therapy to reverse silicosis and its associated pulmonary complications. Lung function continues to deteriorate even when the disease progression is reduced and after the patient is no longer exposed. The median survival rate ranges from 3 to 5 years.^15^ The condition is exacerbated by the undesirable effects of drugs used for treatment.^16^ Lung transplantation remains the only remedy for patients who are refractory to conventional therapy. Currently, pirfenidone and nintedanib are the only approved antifibrotic medications available for clinical use; however, there are limited findings regarding their ability to reduce mycobacterial susceptibility.^17^

Previous research on diosgenin has demonstrated its anti‐inflammatory, anti‐proliferative, and antioxidant properties.^18^ Studies have shown its ability to treat respiratory diseases, as well as liver and cardiac fibrosis. However, the ability of the compound to protect lung tissue from oxidative stress in silica‐induced fibrosis and associated silicosis, or silicotuberculosis, has not received sufficient research attention. This research aims to investigate the efficacy of diosgenin in reducing the susceptibility of rats to *Mycobacterium smegmatis* infection, as well as its antioxidant activity and histological findings after silica‐induced pulmonary fibrosis.

## 2 MATERIALS AND METHODS

### 2.1 In vitro determination of minimum inhibitory concentration (MIC) of diosgenin

The drug susceptibility of *Mycobacterium smegmatis* was investigated using the high‐throughput spot culture growth inhibition assay (HT‐SPOTi) technique.^19^ The aim was to determine the amount of diosgenin required to inhibit the growth of *Mycobacterium smegmatis*. Middle‐brook 7H10 (MB7H10) agar with 0.5% glycerol was autoclaved. Oleic albumin dextrose and catalase (OADC 10% v/v) were added as a supplement. The agar was placed in a water bath at 55° to 60°C to prevent solidification. *Mycobacterium smegmatis mc* ^2^ 155, (ATCC 19420) was inoculated in 10 mL of MB 7H10 and incubated at 37°C for 20–24 h. Microbial growth on the agar was washed with 0.9% (w/v) normal saline solution. A 100 μL aliquot of the washed bacteria was dispensed into a Falcon tube containing 10 mL sterile normal saline. A 1 mL aliquot of the bacterial suspension from the previous tube was transferred into a new Falcon tube containing 9 mL of normal saline. Another 1 mL aliquot of the suspension was further transferred into the last Falcon tube containing 19 mL of normal saline to mark the end of the serial dilution. Drugs/inhibitors (diosgenin, rifampicin, and isoniazid) were weighed and dissolved in 1 mL of 0.3% dimethyl sulfoxide (DMSO) to achieve a stock concentration of 50 mg/mL. Two‐fold serial dilution of the stock solution was carried out in a half‐skirted 96‐well PCR plate as previously reported.^13^

### 2.2 Care of animals

Sprague–Dawley (SD) rats were obtained from the Noguchi Memorial Institute for Medical Research, University of Ghana, Legon. They were housed in the animal house of the Department of Pharmacology at KNUST, Kumasi. The rats were housed in stainless steel cages with soft wood shavings as bedding, fed with a pellet diet (GAFCO, Tema) and water was given ad libitum. The room was maintained at a temperature of 24–28°C and relative humidity of 60–70%, under a 12‐h light–dark cycle. All procedures and techniques used in this study were in accordance with the *National Institute of Health Guidelines for the Care and Use of Laboratory Animals* (8th edition, 2011). Ethical approval (FPPS/008/2018) was obtained from the Department of Pharmacology, Animal Ethics Committee, KNUST, Kumasi, Ghana.

### 2.3 Experimental design

After 7 days of acclimatization, 8‐week‐old SD rats (120–200 g) were randomized into seven groups without exclusion criteria. Ten experimental units of rats were allocated to each group. The experiment was conducted in two stages. Five rats from each group were randomly selected and sacrificed on day 30 to assess silicosis. The remaining five rats in each group received mycobacteria on day 31. Silicosis induction occurred on day 1. Both the silicosis group and the silica plus mycobacterium group, but not the naïve group, were administered diosgenin treatment from day 1 to day 50. Prednisolone and rifampicin were used as standard drugs for treatment of silicosis and mycobacterium, respectively. The entire experiment lasted for 50 days.

Rats were grouped and treated as follows:

Group 1 (Naive) received normal saline 1 mg/kg intratracheally on day 1.
Group 2 (Silica + Myco) received (50 mg/kg) crystalline silica‐only plus 500 × 10^7^ CFU of *Mycobacterium smegmatis* intratracheally on day 1.
Group 3 (Dios 1) received crystalline silica on day 1 and diosgenin (1 mg/kg i.p.) from day 1 to day 50.
Group 4 (Dios 10) received crystalline silica on day 1 and diosgenin (10 mg/kg i.p.) from day 1 to day 50.
Group 5 (Dios 20) received crystalline silica on day 1 and diosgenin (20 mg/kg i.p.) from the first day 1 to day 50.
Group 6 (Pred) received silica on day 1 and prednisolone (10 mg/kg i.p.) from day 1 to 30.
Group 7 (Rif) received silica on day 1 and rifampicin (10 mg/kg i.p.) from day 30 to 50.

Rats were anesthetized with pentobarbitone (40 mg/kg) and 50 mg/kg of crystalline silica was induced intratracheally (50 mg in 100 μL of normal saline for each rat). After silica instillation, the rats were turned upside down immediately to ensure an even distribution of silica in the lungs.^20^, ^21^

### 2.4 Sample collection

On the 30th day following silica induction, the rats were anesthetized with pentobarbitone (40 mg/kg). The chest was gently opened wide, and then both lungs were taken and fixed in 10% formalin for 24 h until the tissue became hard enough for sectioning.

### 2.5 Determination of body weight and lung index

This assay measures the weight of the wet lung in milligrams in relation to the weight of the rat in grams. The pulmonary index is used to indicate the ventilatory status and, thus, the level of pulmonary edema. The rats were weighed before and after the experiment. The weight gain in each category was recorded. After ablation, the extracted lungs were trimmed of foreign tissue, cleaned, and weighed. The pulmonary index was calculated as the proportion of lung weight (mg) to body weight (g) of each animal.

### 2.6 Clinical assessment of silicosis with H&E stain

Rats were assessed for inflammation before infection with mycobacterium. The lungs were dehydrated, embedded in paraffin, and sectioned into 4‐μm‐thick slices. The tissue was then stained with Hematoxylin for 5 min, followed by Eosin (H&E) stain. The slides were viewed under a light microscope (United Medical Laboratories, Riyadh, Saudi Arabia) at a magnification of ×10.^22^

### 2.7 Biochemical analysis

A 4.5 mL tissue homogenizing buffer (pH 7.4) was added to each lung specimen on ice. A tissue homogenizer was used to blend the lung tissues into intracellular components for 15 min. The homogenate was centrifuged at 4000 × g for 20 min, decanted, and assayed.

### 2.8 Growth of mycobacterium culture

MB7H9 broth (0.15 g), glycerol (66 μL) and 0.1% Tween 80 (83 μL) were added to a conical flask, and mixed thoroughly. The solution was autoclaved at 121°C for 10 min and allowed to cool. A 9 mL aliquot of broth was taken into a conical flask, followed by 1 mL of OADC. A 0.1 mL aliquot of the organism was then added and incubated at 37°C for 24 h.^23^

### 2.9 Induction of tuberculosis

For the five remaining rats in each group, each rat was injected intranasally with 500 × 10^7^ CFU/mL of *Mycobacterium smegmatis*. Treatment with diosgenin continued until day 50. On the 20th day post‐infection, the rats were anesthetized by intraperitoneal injection of pentobarbital sodium (40 mg/kg), sacrificed instantly, and the lungs excised. The lung tissues were subjected to Van Gieson stain to differentiate between collagen fibers and smooth muscle.

Lung tissues were fixed in 10% formalin for 36 h, dehydrated and embedded in paraffin. The tissues were sectioned into 4 μm‐thick slices. The sections were stained with Van Gieson stain to assess collagen deposition. The slides were viewed under a light microscope (United Medical Laboratories, Riyadh, Saudi Arabia) at a magnification of ×20.

### 2.10 *M. smegmatis* colony‐forming unit count

To 3.8 g of MB7H10 agar powder, 180 mL of distilled water was added and autoclaved. Sterilized Eppendorf tubes were labeled 1 to 9. A 900 μL of saline was transferred into the tubes. Lung tissues were homogenized in tissue homogenizing buffer, centrifuged and decanted to obtain a bacterial suspension. A 10 mL aliquot of bacterial sample was prepared and vortexed to ensure even distribution. A 100 μL aliquot of the bacteria suspension was aseptically transferred into the labeled tubes. Serial dilution of the bacteria was performed. Each of the Petri dishes was labeled accordingly. A 100 μL of each of the serial dilutions was transferred to the centre of each agar plate and streaked with a spreader. The plate was closed, inverted, and incubated at 37°C.^24^ CFU was calculated as:

$$\begin{array}{c} \text{CFU}=\frac{\text{Number of colonies}\times \text{dilution factor}}{\text{Amount plated}}. \end{array}$$

### 2.11 Evaluation of in vivo antioxidant activity of diosgenin

#### 2.11.1 Superoxide dismutase (SOD) activity

This assay is based on the utilization of the superoxide anion formed as a result of the autoxidation reaction of adrenaline in an alkaline medium. Adrenochrome from adrenaline autoxidation is prevented by SOD.^25^ A 500 μL of homogenized lung tissue was centrifuged in 150 μL of very cold chloroform and 750 μL ethanol (96% v/v) at 2000 g for 20 min. Consecutive increments of 1 mL carbonate buffer (0.1 M, pH 10.2) and 0.5 mL EDTA (0.6 mM) were added to 500 μL of supernatant aliquot. A 0.05 mL of (1.3 mM) adrenaline was then added to generate adrenochrome. A blank was prepared, which excluded the tissue homogenate. A 150 μL of the resultant was pipetted onto the microplate. The values for absorbance were measured at 480 nm by a Synergy H1 Multi‐Mode Reader (BioTek Innovations, Winooski, USA). The percentage hindrance of adrenaline autoxidation was determined as:

$$\text{Inhibition}=\left(\frac{{\text{Absorbance}}_{\text{test}}-{\text{Absorbance}}_{\text{blank}}}{{\text{Absorbance}}_{\text{test}}}\right)\times 100.$$

Superoxide dismutase action was measured in units per mg protein, where a unit is the amount of the enzyme that inhibited 50% autoxidation of adrenaline at 25°C; determined from the equation:

Units of SOD activity/mg protein = (% inhibition)/(50 × weight of protein).

#### 2.11.2 Catalase (CAT) activity

This assay is based on the reduction of dichromate in acetic acid to chromic acid when heated in the presence of hydrogen peroxide. The chromic acetate produced is then measured calorimetrically.^26^ The assay mixture, containing 0.4 mL of H_2_O_2_ (1.18 M) and 1 mL of phosphate buffer (0.01 M, pH 7.0), was added to 0.1 mL of tissue homogenate and incubated for 5 min at room temperature. A volume of 2 mL of dichromate‐acetic acid (containing three parts of glacial acetic acid and 1 part of 5% potassium dichromate) was added to end the response. A 150 μL aliquot of the resultant mixture was transferred into a 96‐well microplate. The absorbance was measured using spectrophotometry at a wavelength of 620 nm. CAT activity was calculated based on the extinction coefficient of H_2_O_2_, 39.4/M/cm at 620 nm, and expressed as a unit per milligram protein. One unit of the CAT enzyme is expected to hydrolyze 1 mmol of H_2_O_2_ per min, at pH = 7 and 25°C.

$$\text{Unit of}\;\left(\mathrm{CAT}\;\text{activity}/\left(\mathrm{mg}\;\text{protein}\right)\right)=\frac{\left(\text{Absorbance}\left(620\;\mathrm{nm}\right)\right)}{\left(39.4\times \text{weight of protein}\right)}\times 1000.$$

#### 2.11.3 Lipid peroxidation assay

Lipids have unique structural and functional roles in cell membranes. Disturbing this role through covalent binding and oxidation can lead to cell death. The extent of damage is determined by how much malondialdehyde (MDA) is produced. This assay illustrates the extent of oxygen‐driven damage in the thiobarbituric acid (TBA) assay, as indicated by the color change. The quantity of MDA was measured as previously described by Heath and Parker.^27^ To 1 mL of tissue homogenate, 3 mL of a mixture of 20% TCA in 0.5% TBA was added. The mixture was warmed at 95°C for half an hour, and allowed to cool. The sample was then centrifuged at 5000 × g for 10 min. Absorbance was measured at 532 nm and then at 600 nm to correct for nonspecific absorbance. The molar extinction coefficient of the MDA‐TBA product, 155/mM/cm was used to quantify the amount of MDA formed.

### 2.12 Statistical analysis

GraphPad Prism version 8 was used for all statistical analyses. Data with two independent variables were analyzed using two‐way ANOVA followed by Dunnett's post hoc test, whereas those with a single independent variable were analyzed using one‐way ANOVA. Results were presented as mean ± SEM. *p* < 0.05 was considered statistically significant.

## 3 RESULTS

### 3.1 In vitro determination of the minimum inhibitory concentration of diosgenin

The half‐maximal inhibitory concentration (IC50) of diosgenin was determined to be 0.006043 μg/mL, with a correlation coefficient of 0.86 (Table 1).

**TABLE 1:** Half‐maximal inhibitory concentration (IC_50_) of diosgenin, isoniazid and rifampicin against *Mycobacterium smegmatis*.

Drug	IC_50_ (μg/mL)
Diosgenin	0.006043
Isoniazid	0.1192
Rifampicin	52.55

### 3.2 Body weight changes

There was a significant improvement in the body weight of rats treated with diosgenin compared to the silica‐only group. The silicosis group weighed an average of 128 g before the experiment and 128.7 g at the end of the experiment. In the silica plus mycobacterium group, the 1 mg/kg diosgenin‐treated group weighed 155.1 g at the beginning and 162 g at the end of the experiment. The 10 mg/kg diosgenin weighed 150.4 g before and 159 g at the termination of the experiment. The 20 m/kg group weighed 190.3 g and 198 g before and after the experiment, respectively. Treatment with diosgenin at all doses (1, 10, and 20 mg/kg) resulted in significant weight gain, suggesting an improvement in overall metabolic status and immune function.

### 3.3 Lung index

**FIGURE 1:**
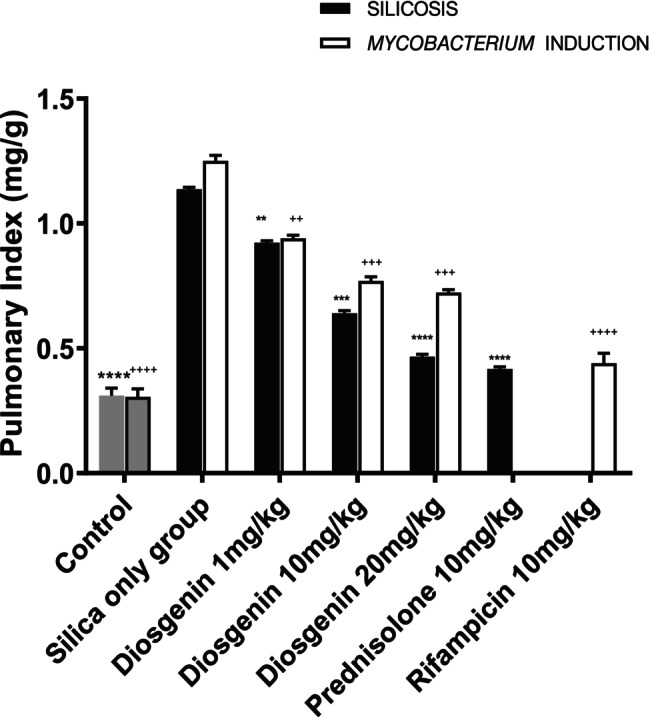
Effect of diosgenin on the pulmonary index. Diosgenin reduced lung weights in both the silicosis and the silica plus *Mycobacterium* groups. Data are expressed as mean ± SEM. *n* = 10, one‐way ANOVA and Dunnett's post hoc test. ****p < 0.0001, ***p < 0.001, **p < 0.01 vs. silica group; ^*++++*^ *p <* 0.0001, ^*+++*^ *p <* 0.001, ^*++*^ *p < 0.01* vs. *Mycobacterium* group.

The lung weight of rats after the silica challenge increased to a mean value of 0.8260 ± 0.02084 mg/g (*p* < 0.0001) compared to the naive control rats. Diosgenin administration reduced the mean weight to 0.2133 ± 0.02084 mg/g (*p <* 0.01), 0.4967 ± 0.02084 (*p <* 0.001), 0.6700 ± 0.02084 mg/g (*p* < 0.0001) at doses of 1, 10 and 20 mg/kg, respectively, with an *F* value of 474.3 mg/g. After introduction of silica followed by *Mycobacterium*, the mean difference rose to 0.9440 ± 0.03504 mg/g (*p <* 0.0001) compared to the naive control rats. Rifampicin significantly (*p <* 0.05) reduced the lung weight to a mean of 0.8100 ± 0.03504 mg/g (*p <* 0.0001). Diosgenin administration reduced the total lung weight (mg/g) to 0.3100 ± 0.03504 (*p <* 0.01), 0.4800 ± 0.03504 (*p <* 0.001), 0.5265 ± 0.03504 mg/g (*p <* 0.001) at doses of 1, 10 and 20 mg/kg, respectively, with an *F* value of 188.8 (Figure 1). In a previous study, inflammation was found to lead to skeletal muscle wasting and weight loss.^28^

### 3.4 Diosgenin reduced the mycobacterium colony‐forming units (CFUs)

**FIGURE 2:**
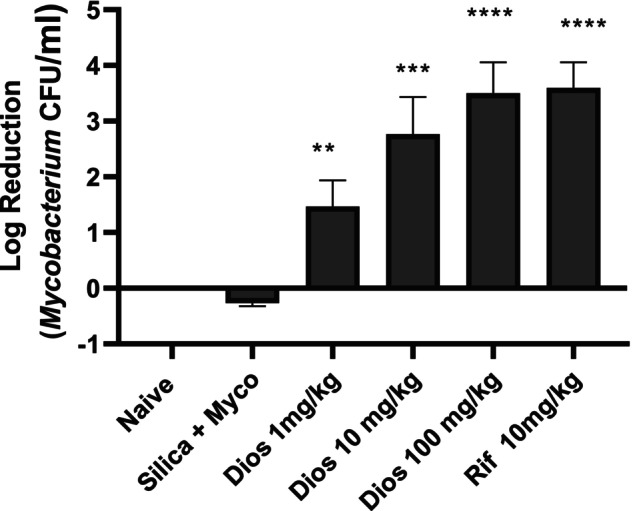
Effect of diosgenin on Mycobacterium colony‐forming units. Diosgenin reduced the number of viable bacteria in the lung tissue homogenate. The data are expressed as mean ± SEM. *n* = 10, one‐way ANOVA and Dunnett's post hoc test. **p < 0.01, ***p < 0.001, ****p < 0.0001 vs. *Mycobacterium* induction.

Diosgenin significantly reduced the number of viable bacteria in the lung tissue homogenate after infection with *Mycobacterium smegmatis* to 10.81 ± 1.748 mycobacteria CFU/mL (*p* < 0.0001), compared with the control. Addition of 10 mg/kg rifampicin resulted in significant growth inhibition of bacterial cells with a mean difference of 9.130 ± 1.748 (*p <* 0.0001). Diosgenin inhibited the growth of *Mycobacterium smegmatis* to 1.030 ± 1.748 (*p =* 0.9633), 2.140 ± 1.748 (*p =* 0.6257) and 3.370 ± 1.748 mycobacterium CFU/mL (*p =* 0.2342) at doses of 1, 10 and 20 mg/kg, respectively, with an F value of 13.14 (Figure 2).

### 3.5 Antioxidant effect of diosgenin

#### 3.5.1 Diosgenin elevated SOD level

The antioxidant effect of diosgenin was significantly reduced after the silica challenge, with a mean difference of −0.1992 ± 0.01363 U/mg protein compared to the naive control group (*p < 0.05*). Administration of 10 mg/kg prednisolone resulted in a decline of −0.122 ± 0.01363 U/mg protein (*p <* 0.0001). Diosgenin increased the activity of superoxide dismutase by mean values of −0.1355 ± 0.01363 (*p <* 0.0001), −0.1451 ± 0.01363 (*p <* 0.0001) and − 0.1970 ± 0.01363 U/mg protein (*p <* 0.0001) at doses of 1, 10 and 20 mg/kg, respectively.

**FIGURE 3:**
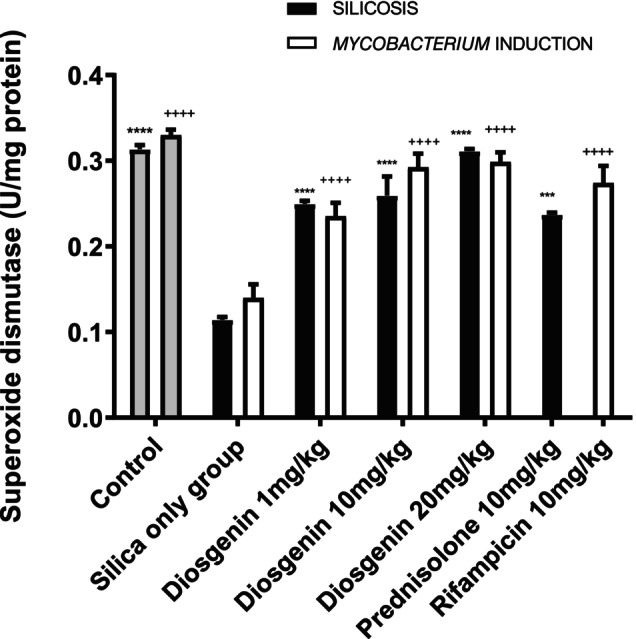
Effect of diosgenin on superoxide dismutase activity. The data are expressed as mean ± SEM. *n* = 10, one‐way ANOVA and Dunnett's post hoc test. ****p < 0.0001 vs. silica only group; ^*++++*^ *p <* 0.0001 vs. *Mycobacterium* induction.

The *Mycobacterium* plus silica group showed significant levels of enzyme activity, with a mean difference of −0.1000 ± 0.001825 U/mg protein (*p <* 0.05) (Figure 3). Rifampicin (10 mg/kg) resulted in significant enzyme activity, with a mean difference −0.2641 ± 0.001825 (*p <* 0.05). Similarly, diosgenin administration led to improvements in superoxide dismutase activity of −0.09527 ± 0.001825 (*p <* 0.0001), −0.152 ± 0.001825 (*p <* 0.0001) and − 0.2327 ± 0.001825 (*p <* 0.0001) at doses of 1, 10 and 20 mg/kg, respectively, with an *F* value of 20.65 (Figure 3).

#### 3.5.2 Diosgenin elevated CAT levels

The ability of catalase to break down hydrogen peroxide was significantly reduced after the silica challenge compared with the control group, with a mean difference of −0.5213 ± 0.05311 U/mg protein (*p <* 0.0001) Administration of 10 mg/kg prednisolone increased catalase activity by −1.493 ± 0.05311 U/mg protein (*p <* 0.0001). Diosgenin also increased the activity of catalase by mean values of −0.1506 ± 0.0531 (*p <* 0.0001), −0.6329 ± 0.05311 (*p =* 0.431) and − 0.9318 ± 0.05311 U/mg protein (*p <* 0.0001) at doses of 1, 10 and 20 mg/kg, respectively, with an *F* value of 209.0.

**FIGURE 4:**
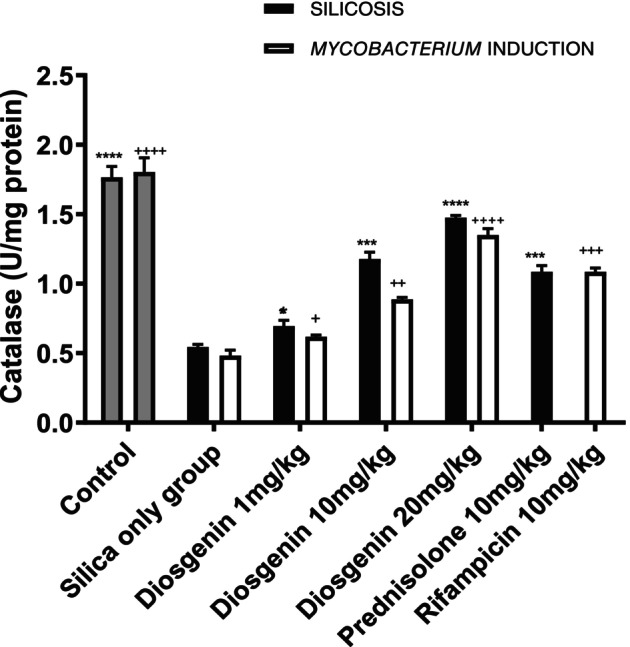
Effect of diosgenin on catalase activity. The data are expressed as mean ± SEM. *n* = 10, one‐way ANOVA and Dennett's post hoc test *p < 0.1, ***p < 0.001 vs. silica‐only group; ^+^ *p =* 0.01, ^*+++*^ *p <* 0.001 vs. *Mycobacterium* induction.

The *Mycobacterium* plus silica group recorded significant levels of enzyme activity, with a mean difference of −0.5723 ± 0.04207 (*p <* 0.0001). Rifampicin (10 mg/kg) resulted in considerable enzyme activity, with a mean difference of −0.9594 ± 0.04207 (*p <* 0.0001). Diosgenin administration led to improvements in antioxidant activity of −0.1369 ± 0.04207 (*p =* 0.0181), −0.4052 ± 0.04207 (*p <* 0.01) and − 0.8689 ± 0.04207 U/mg protein (*p <* 0.0001) at doses of 1, 10 and 20 mg/kg, respectively, with an F value of 20.65 (Figure 4).

#### 3.5.3 Diosgenin reduced MDA levels

Oxidative stress status was associated with a state of inflammation, as indicated by a significant elevation of malondialdehyde levels (65.96 ± 3.869 nmol/mg protein) compared to the naive control group (*p <* 0.05). Prednisolone (10 mg/kg) reduced MDA levels to 127.7 ± 3.869 nmol/mg protein (*p* < 0.0001) compared with the silica‐only group. Diosgenin significantly reduced the mean MDA levels by 24.17 ± 3.869 (*p* < 0.0001), 34.10 ± 3.869 (*p <* 0.01) and 141.1 ± 3.869 nmol/mg protein (*p <* 0.0001) at doses of 1, 10 and 20 mg/kg, respectively. The *F* value was 442.9.

**FIGURE 5:**
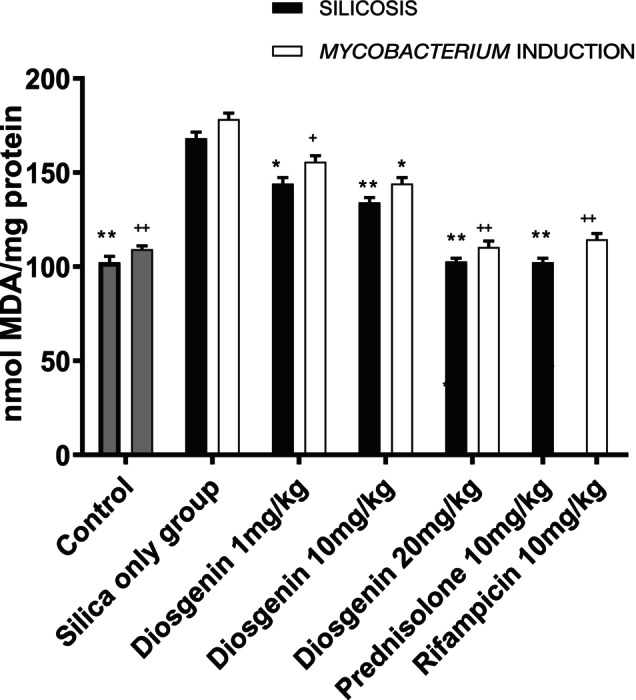
Effect of diosgenin on lipid peroxidation. The data are expressed as mean ± SEM. *n* = 10, one‐way ANOVA, and Dunnett's post hoc test. **p < 0.01, *p < 0.1 vs. silica group; ^*++*^ *p <* 0.01, ^+^ *p <* 0.1 vs. mycobacterium induction.

The *Mycobacterium* plus silica group showed significant levels of MDA, with a mean difference of 69.13 ± 5.261 nmol/mg protein (*p <* 0.0001). Rifampicin (10 mg/kg) resulted in a significant reduction in oxidative stress markers, with a value of 63.90 ± 5.261 nmol/mg protein (*p <* 0.0001). Similarly, diosgenin administration reduced MDA levels to 22.64 ± 5.261 (*p <* 0.01), 34.27 ± 5.261 (*p <* 0.01) and 67.98 ± 5.261 nmol/mg protein (*p <* 0.01) at doses of 1, 10 and 20 mg/kg, respectively, with a reported *F* value of 59.01 (Figure 5).

### 3.6 Histological findings

#### 3.6.1 Microscopic findings with H&E stain

**FIGURE 6:**
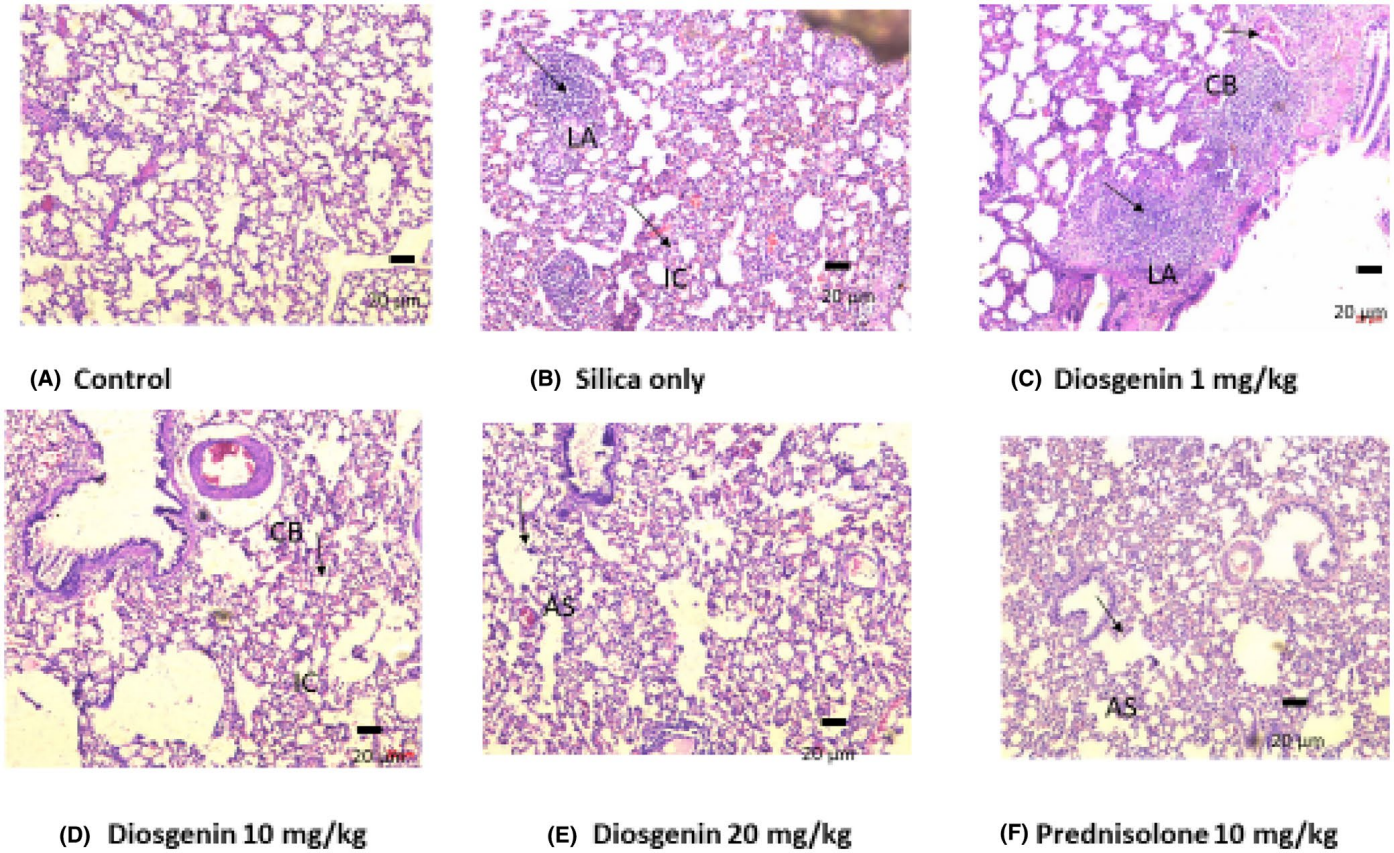
Representative photomicrograph of a section of lung tissue (H&E stain) (A) control; (B) silica plus *Mycobacterium*; (C) 1 mg/kg diosgenin; (D) 10 mg/kg diosgenin; (E) 20 mg/kg diosgenin; (F) prednisolone 10 mg/kg. AS, alveoli space; CB, congested blood vessels; IC, inflammatory; LA, lymph activation. Magnification ×10.

Hematoxylin and Eosin stain were used to differentiate between types of connective tissue and fibers. Hematoxylin is a basic dye that stains the acidic component of wax‐cleared tissue, while the acidic Eosin stains the fundamental component of the tissue. The normal control (A) is lung tissue composed microscopically of patent alveolar spaces. Blood vessels are not congested. There is no infiltration of inflammatory cells, typical of normal lung tissue. In Figure 6B, the lung tissue shows extensive damage to the parenchyma. There is evidence of massive necrotic debris (pus formation). The underlying lymph nodes show evidence of reactive sinus hyperplasia. In Figure 6C, diosgenin administration at a dose of 1 mg/kg shows lymph node activation and infiltration of inflammatory cells. In Figure 6D, the 10 mg/kg dose of diosgenin depicts mild to moderate lung parenchyma destruction with patent alveoli. In Figure 6E, 20 mg/kg diosgenin and 10 mg/kg prednisolone show moderately healed lung parenchyma. The alveoli spaces are patent. Findings are most consistent with a healed lung parenchyma (Figure 6).

#### 3.6.2 Microscopic findings with Van Gieson stain

**FIGURE 7:**
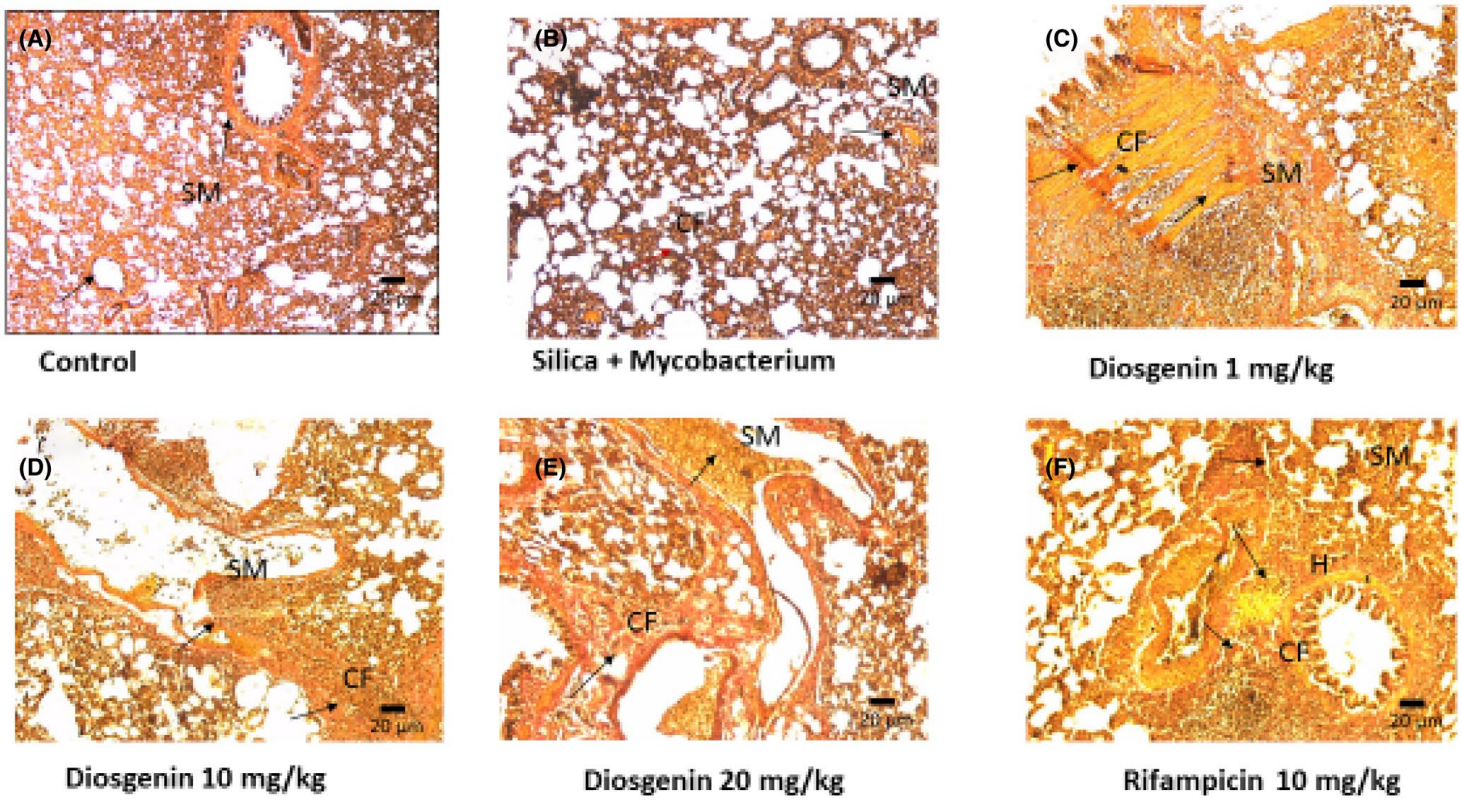
Representative photomicrograph of a section of lung tissue (Van Gieson stain) (A) control; (B) silica plus *Mycobacterium*; (C) 1 mg/kg diosgenin; (D) 10 mg/kg diosgenin; (E) 20 mg/kg diosgenin; (F) 10 mg/kg rifampicin. CF, collagen fibers; H, hyalin; SM, smooth muscle. Magnification ×10.

Van Gieson stain was used to differentiate collagen fibers from muscle and red blood cells. The collagen fiber is stained red and smooth, and the striated muscle is stained yellow. The control showed standard lung architecture with ciliated thin epithelium lining the terminal bronchi. Silica plus *Mycobacterium* showed extensive infiltration of necrotic debris into the alveolar space. Terminal bronchi are shrunken with eroded cilia. At 1 mg/kg, diosgenin shows infiltration of cells into the alveolar space. Collagen fibers are visible. Administration of 20 and 10 mg/kg diosgenin shows moderately healed lung parenchyma with few remnants of inflammatory cells (Figure 7).

## 4 DISCUSSION

Silicosis is a work‐related lung disease caused by excessive exposure to silica. It results in irreversible and serious health effects in silica‐exposed workers. Silica‐induced fibrosis remains the most reliable and frequently used experimental model of silicosis.^21^, ^29^ In this regard, we sought to investigate the ability of diosgenin to inhibit the growth of *Mycobacterium smegmatis* and its antioxidant potential in the treatment of silica‐induced tuberculosis. The study was supported by a histological examination of the lung tissue, which employed the use of Hematoxylin and Eosin and Van Gieson stains.

According to the results in Table 1, diosgenin inhibited the growth of *Mycobacterium smegmatis* with an IC_50_ value of 0.006043 μg/mL (*r* = 0.86). Interestingly, the mainstream standard drugs recorded IC_50_ values of 0.1192 and 52.55 μg/mL for isoniazid and rifampicin, respectively. Hence, our findings (Table 1) suggest diosgenin could be more potent than isoniazid and rifampicin. In a previous study,^30^ diosgenin exhibited similar antibacterial effects through inhibition of biofilms, the means by which bacteria become resistant to antimicrobial agents.^31^ The mechanism by which diosgenin exerts this antibacterial effect is still unclear, but it may involve disrupting bacterial cell wall synthesis, inhibiting biofilm formation, or modulating immune responses. A significant improvement can be made by studying the synergistic ability of diosgenin with one of the mainstream antitubercular drugs. Diosgenin could be developed as a potential adjunct to the current antitubercular agents in the fight against multidrug‐resistant tuberculosis.

Weight loss is a hallmark of chronic inflammation and tuberculosis. Diosgenin's effect may be linked to its ability to modulate inflammatory pathways and reduce oxidative stress, preventing excessive muscle wasting. Previous studies have shown that diosgenin exhibits anti‐inflammatory properties by inhibiting pro‐inflammatory cytokines (TNF‐α, IL‐6) and reducing oxidative stress.^32^

Loss of pulmonary membrane integrity was indicated by the infiltration of cells and fluid into the lung parenchyma. Increases in lung weight and the ratio of wet to dry lung weights and were used to indicate pathological changes in the lung.^33^ In a previous study, silica induction led to oxidative stress and mitochondrial membrane dysfunction in lung epithelial cells.^34^

The ability of superoxide dismutase to break the harmful oxygen molecule into a less damaging form is an essential factor in immune defense. The increase in the antioxidant activity of diosgenin may be due to its ability to activate the endogenous antioxidant level.^35^ In similar studies,^36^ the observation was supported by a relative reduction in oxidative damage in a feed containing diosgenin. Rats supplemented with 0.1 and 0.5% diosgenin powder in a feed showed enhanced resistance to DNA damage and suppressed weight gain.

Catalase is an enzyme present in nearly all living organisms. It breaks down hydrogen peroxide produced as a result of cellular respiration into water and oxygen.^37^ Antioxidant activity was quantified after silica and *Mycobacterium* administration. An increase in enzyme activation corresponded to a reduction of mucus accumulation in the lung tissue. This finding is consistent with previous work, where a 40 mg/kg dose of diosgenin had a pronounced effect on enzyme activation compared to the 20 mg/kg dose in the present study. The difference may be due to the variable length of diosgenin administration and the dosage. Administration of diosgenin is linked to rebuilding endogenous antioxidant capacity after silica lung damage.^38^, ^39^

MDA levels were quantified to determine the extent of damage caused by the oxidative degradation of unsaturated fatty acids in the cell membrane. Diosgenin administration resulted in a significant reduction in MDA levels, indicating its potential to inhibit mitochondrial membrane impairment. Our findings suggest that using diosgenin as an antioxidant supplement may be useful in protecting against oxidative stress in patients with compromised immune defenses due to mitochondrial membrane irritation. The above result is consistent with previous studies^39^, ^40^ in which 40 mg/kg of diosgenin led to a decline in the level of MDA in impaired mitochondrial oxidative stress. In another study,^32^ diosgenin defended the bio membranes of mice fed a high‐fat diet through a decline of lipid peroxidation.

Massive destruction of the lung parenchyma was observed in the Van Gieson‐stained tissue compared to the H&E. From Figures 6 and 7, it can be deduced that the wall of the granuloma formed by cellular infiltration of infected immune cells got raptured, releasing the contents into the lymphatic vessel which led to activation of the lymph node. In a previous study, diosgenin administration in H&E‐stained sciatic nerve restored tissue damage through the antioxidant defense system.^41^ In our study, the maximum dose resulted in a significant improvement in lung healing. The degree of inflammation in the 20 mg/kg group was significantly reduced (Figure 6). To achieve the maximum healing effect of diosgenin, it is therefore recommended that the treatment be continued for more than 50 days. The number of inflammatory cells was not quantified, and the data do not include the type of collagen fibers involved.

In brief, silica‐induced damage to alveolar macrophages and mycobacterial infection may play a crucial role in the progression of oxidative damage in the lung parenchyma. Diosgenin administration led to a significant improvement in pulmonary function, characterized by a decline in oxidative damage and inhibition of mycobacterial growth, and therefore has the potential to be developed as a drug to combat silicotuberculosis.

## 5 CONCLUSION

Diosgenin attenuated lung injury through inhibition of *Mycobacterium smegmatis* growth, a decline in malondialdehyde levels and elevation of antioxidant enzymes. The compound, therefore, represents an interesting alternative means of treating lung fibrosis and can serve as a useful lead compound for the development of a drug to tackle silica‐induced tuberculosis.

## AUTHOR CONTRIBUTIONS

**Williams Asamoah Adu:** Conceptualization; formal analysis; methodology. **Paul Poku Sampene Ossei:** Project administration; resources; supervision. **Cynthia Amaning Danquah:** Project administration; resources; supervision. **Selase Ativui:** Conceptualization; methodology. **Michael Ofori:** Formal analysis; validation. **George Owusu:** Validation; visualization; writing – review and editing.

## FUNDING INFORMATION

None.

## CONFLICT OF INTEREST STATEMENT

The authors declare no competing interest.

## ETHICS STATEMENT

Ethical approval (FPPS/008/2018) was obtained from the Department of Pharmacology, Animal Ethics Committee, KNUST, Kumasi, Ghana.

## Data Availability

The data that supports the findings of this study are available in the supplementary material of this article.
